# Very Low Uptake in Workplace Semen Analysis Research: Formative Web-Based Cross-Sectional Follow-Up Survey Distinguishing Employees With Self-Reported Unawareness From Aware Nonparticipants

**DOI:** 10.2196/90788

**Published:** 2026-07-13

**Authors:** Ayumi Nakazono, Kosuke Kojo, Tomoko Oguri, Hirokazu Nosato, Atsushi Ikeda, Toshiyuki Kakinuma, Hiroyuki Nishiyama, Hideki Takeshita, Tatsuya Takayama, Kaoru Yanagida, Osamu Tsutsumi

**Affiliations:** 1 Center for Human Reproduction, International University of Health and Welfare Hospital, Nasushiobara, Tochigi, Japan; 2 Tsukuba Clinical Research & Development Organization (T-CReDO), University of Tsukuba, Tsukuba, Ibaraki, Japan; 3 Department of Urology, Institute of Medicine, University of Tsukuba, 1-1-1 Tennodai, Tsukuba, Ibaraki, 305-8575, Japan, 81 29-853-3223, 81 29-853-8854; 4 Research Institute of Science for Safety and Sustainability (RISS), National Institute of Advanced Industrial Science and Technology (AIST), Tsukuba, Ibaraki, Japan; 5 Artificial Intelligence Research Center (AIRC), National Institute of Advanced Industrial Science and Technology (AIST), Tsukuba, Ibaraki, Japan; 6 Department of Obstetrics and Gynecology, Yokohama City Seibu Hospital, St. Marianna University School of Medicine, Yokohama, Kanagawa, Japan; 7 Department of Urology, International University of Health and Welfare Hospital, Nasushiobara, Tochigi, Japan; 8 Center for Human Reproduction and Gynecologic Endoscopy, Sanno Hospital, Minato-ku, Tokyo, Japan

**Keywords:** semen analysis, male reproductive health, occupational health, workplace research, recruitment, survey methodology, informed consent, privacy, preconception care

## Abstract

**Background:**

Very low uptake in workplace semen analysis research is difficult to interpret, particularly in employer-adjacent settings, where nonparticipation may reflect limited recruitment reach, limited understanding of the occupational rationale, low perceived relevance, or procedure-related concerns.

**Objective:**

This post hoc formative study described self-reported awareness of a parent workplace semen analysis study as an indicator of effective recruitment reach, reported reasons for nonparticipation under the implemented survey condition, and design issues for exposure-defined workplace reproductive health research.

**Methods:**

In April-May 2025, we conducted an anonymous web-based cross-sectional follow-up survey among male employees in Japan who had been eligible for, but had not completed, a parent workplace semen analysis study. The parent study invited approximately 2000 male employees from 3 companies between November 2024 and January 2025; 6 completed the protocol. The follow-up survey invited approximately 900 male employees from 1 company. Part 1 assessed awareness, reasons for nonparticipation, interest in male reproductive health information, and general openness to future related research. Optional Part 2 assessed age, knowledge, concerns, expected reactions, and willingness under simplified conditions. Responses were summarized descriptively using Wilson 95% CIs; no hypothesis testing was performed.

**Results:**

We analyzed 108 submitted questionnaires; 83 respondents completed Part 2. Overall, 74/108 (68.5%; 95% CI 59.3‐76.5) respondents reported no awareness of the parent study. Among unaware respondents, 68/74 (91.9%; 95% CI 83.4‐96.2) selected “did not know the study existed.” Among aware nonparticipants, the most frequent reasons were perceived irrelevance and resistance to collecting semen (each 9/34, 26.5%; 95% CI 14.6‐43.1), embarrassment or reluctance (8/34, 23.5%; 95% CI 12.4‐40), and hassle (7/34, 20.6%; 95% CI 10.3‐36.8). In Part 2, anxiety about unfavorable results was reported by 52/83 (62.7%; 95% CI 51.9‐72.3) respondents, concerns about collection location or privacy protection by 48/83 (57.8%; 95% CI 47.1‐67.9), and self-reported resistance by 42/83 (50.6%; 95% CI 40.1‐61.1). Under simplified conditions, 36/83 (43.4%; 95% CI 33.2‐54.1) respondents indicated willingness to undergo semen analysis.

**Conclusions:**

Very low uptake in this employer-adjacent semen analysis study was not interpretable as a single phenomenon. This post hoc formative process evaluation identified limited awareness, suggesting limited effective recruitment reach under the implemented procedures, and characterized the reason profile among aware nonparticipants, including low perceived relevance and semen collection–related concerns. Rather than identifying primary causal determinants of nonparticipation, the findings support a bounded recruitment-methodological interpretation and highlight recruitment-cascade components for prospective measurement: objective exposure to recruitment materials, information access, understanding of the occupational rationale, voluntary postinformation declination, privacy concerns, logistical burden, and specimen-return completion. Informed acceptability after occupational reproductive-hazard education should be evaluated in future designs that include such education and comprehension assessment.

## Introduction

Recruiting sufficient participants is challenging in many epidemiological studies, particularly when the topic is sensitive or the procedure is burdensome [1]. Similar problems have been reported in studies of serious disease [2], invasive research [3,4], and sensitive interview–based studies, particularly among women [5]. This challenge may be even greater in male fertility research [6] because semen analysis can be associated with embarrassment, stigma, masculinity-related concerns, and anxiety about the results [7,8].

Semen quality is increasingly studied not only as an indicator of fertility but also as a marker of broader male health. Poor semen parameters have been associated with increased risks of mortality [9], cardiovascular disease [10], diabetes [11], and certain cancers [12]. Workplace-based reproductive health research also has a clear scientific rationale because reproductive hazards may arise from metals, solvents, pesticides, ionizing radiation, heat, psychosocial stressors, and shift work [13-16]. In occupational studies, exposures may be higher and easier to characterize than in the general population [17,18]. The European Asclepios program combined questionnaire-based time-to-pregnancy data with semen studies to examine specific occupational exposures [19]. Japanese occupational semen studies have also been conducted, including a study of insecticide sprayers [20]. Pesticide-related effects on human sperm have remained an important concern in later reviews [21]. This broader perspective is consistent with growing interest in male preconception care [22,23]. In this study, the workplace was used as a practical exposure-related research setting rather than as a national screening program or an employer wellness screening activity. The parent study focused on occupational and environmental factors, including shift work, and semen analysis was offered only as a voluntary research procedure. Because recruitment was broad and exploratory, the perceived personal relevance of semen testing likely varied across invited workers.

At the same time, semen analysis in asymptomatic workers raises special ethical concerns. Participation must remain fully voluntary, and declining testing is a legitimate choice [24-26]. In employer-adjacent settings, particular attention must be paid to privacy, nondiscrimination, and power imbalances [27,28]. Research ethics guidance also emphasizes the importance of distinguishing potential individual benefits from broader scientific benefits [29], communicating risks and benefits clearly [30,31], and respecting the option not to receive individual results [32].

Low fertility knowledge may reduce perceived personal relevance, but information alone may not overcome emotional or procedural barriers. Men often have limited knowledge of male infertility [33,34], and public messages about fertility may be resisted when they are perceived as stigmatizing [35] or disconnected from everyday concerns [36]. The perceived relevance of fertility-related testing may also vary in contemporary Japan, where reproductive intentions have become more diverse [37-40]. Thus, low perceived relevance in a broad exploratory workplace invitation should not be assumed to represent either an informed judgment after occupational reproductive health education or a stable attitude toward semen testing.

Our team previously conducted a voluntary workplace-based study that combined questionnaires, semen analysis, and urinalysis among male manufacturing employees. Despite free testing and a home-collection protocol, only 6 of approximately 2000 invited employees completed the parent protocol. This very low uptake raised a methodological and ethical interpretive problem: uptake alone could not show whether employees had not been effectively reached, had noticed the recruitment but not understood the occupational or reproductive health rationale, or had chosen not to participate because of low perceived relevance or procedure-related concerns. Recruitment literature similarly cautions that poor enrollment may reflect design, communication, setting, and participant-level factors rather than participant willingness alone [4].

We therefore conducted an anonymous web-based follow-up survey among employees who had been eligible for, but had not completed, the parent workplace semen analysis study. The aim was to describe self-reported awareness of the parent study as an indicator of effective recruitment reach and to summarize reported reasons for nonparticipation under the recruitment and survey procedures actually implemented. The study functioned as a post hoc formative recruitment-process assessment under the implemented recruitment and information conditions, with its inferential scope centered on recruitment methodology, reported reasons, and design implications for future exposure-defined workplace reproductive health research, rather than on population-level attitudes toward semen analysis, causal determinants of nonparticipation, or informed acceptability after occupational reproductive-hazard education.

## Methods

### Study Design and Reporting Standards

This study was designed as a formative, anonymous, web-based cross-sectional survey of male employees who had been eligible for, but had not completed, a workplace study involving semen analysis. It is reported in accordance with the CHERRIES (Checklist for Reporting Results of Internet E-Surveys) [41] and the CROSS (Consensus-Based Checklist for Reporting of Survey Studies) [42]. The completed checklists are provided in Checklist 1 and Checklist 2.

### Ethical Considerations

The study was conducted in accordance with the Declaration of Helsinki and was approved by the Institutional Ethics Committee as an amendment to the original occupational reproductive health project (approval number 24-TA-055). The approved amendment materials included the follow-up survey protocol, recruitment procedure, participant-facing explanation, and electronic consent text, complete Japanese questionnaire, and procedures for anonymous data collection and data management. After viewing the participant-facing explanation, respondents who proceeded to and submitted the questionnaire were considered to have provided electronic informed consent.

The participant-facing explanation stated that responses would be used to analyze research data and to improve future survey methods. This paper reports the anonymous follow-up survey data within this approved consent framework. Specifically, the paper uses the data as a bounded formative recruitment-process assessment of the recruitment and information conditions actually implemented in the parent workplace semen analysis study. The inferential scope of this paper is deliberately restricted. The paper does not introduce a new participant-facing objective, a new data-collection procedure, or a new use of identifiable participant information. It does not report the data as if respondents had received occupational reproductive health education, completed a comprehension assessment, or answered postinformation willingness or declination items.

Participation was strictly voluntary, and no financial or other incentives were provided. The survey was administered via Google Forms, and access to the raw dataset was restricted to the investigators. No personal identifiers were collected, and no identifiable or individual-level data were shared with the cooperating companies. IP addresses and platform server logs were not accessed. The cooperating companies provided access to recruitment settings only and had no role in protocol development, questionnaire design, access to raw data, data analysis, data interpretation, manuscript preparation, or the decision to submit the manuscript.

This follow-up survey was designed to describe nonparticipation under the recruitment and information conditions actually implemented. It was not an occupational exposure assessment, workplace screening program, or educational intervention. The questionnaire did not collect job-specific or chemical-specific exposure data, did not assess workers’ awareness of occupational reproductive hazards, and did not provide an occupational reproductive-hazard education module. Therefore, the findings should be interpreted as descriptive responses under the implemented survey condition, not as workers’ informed attitudes after balanced occupational reproductive health education.

To confirm the ethical scope of this bounded descriptive reporting, we submitted a protocol clarification concerning the present use of the anonymous follow-up survey data to the institutional ethics committee. The Committee determined that this use was within the scope of approval number 24-TA-055 and that reconsent was not required for the anonymous dataset. This determination was consistent with the combined facts that individual respondents cannot be reidentified or recontacted from the analytic dataset, the current reporting does not extend beyond the purposes communicated to participants, and the bounded descriptive analysis imposes no additional risk or burden on respondents.

### Setting and Recruitment (Parent Study Context)

The parent study was conducted from November 2024 to January 2025 and examined the effects of environmental exposure and 3-shift work on fertility in working-age men. Approximately 2000 eligible male employees from 3 cooperating companies in the medical care region of the International University of Health and Welfare Hospital were targeted. Recruitment relied mainly on passive workplace publicity through flyers and digital signage with QR codes linking to the application form, although the number of employees who actually viewed these materials was not tracked. Participants were offered free semen analysis. Samples were collected at home but had to be submitted to the laboratory in person, so the protocol reduced on-site collection demands without eliminating the privacy and logistical burden of semen collection and transport. These issues have been identified as central design challenges in occupational semen research [17,43]. Details of the parent study are summarized in Multimedia Appendix 1.

The minimum target for the parent study was 30 participants, set as a pragmatic feasibility threshold for an exploratory pilot analysis rather than on the basis of a formal power calculation. Despite efforts to recruit beyond this minimum, only 6 individuals completed the parent study. We therefore conducted the present survey in April and May 2025 among approximately 900 male employees from 1 cooperating company. Invitations were distributed as business card–sized flyers during routine health checkups and included a QR code (DENSO WAVE Incorporated) linking directly to a Google Form.

To protect anonymity and reduce concerns about workplace surveillance, invitations were distributed offline, and the questionnaire was accessed directly via QR code without prospectively configured web analytics or server-log tracking. In accordance with CHERRIES guidance, no formal view rate or participation rate is reported because the numbers of landing-page visitors and questionnaire starts were unavailable. Instead, we report the number of analyzable submitted questionnaires and the Part 2 completion proportion among analyzable submitted questionnaires. Because eligibility criteria were broad, some respondents were outside the typical preconception target group, and the perceived relevance of semen analysis likely varied across the sample.

### Survey Development, Structure, and Measures

The questionnaire was developed by the research team based on experiences from the parent study and previous literature on barriers to male participation in fertility research [6,7] and research participation more broadly [44]. Draft items underwent multidisciplinary expert review for face validity and clarity, but formal pilot testing and cognitive interviewing were not performed. The questionnaire should therefore be considered a study-specific preliminary instrument for formative description rather than a validated measure of underlying constructs.

The survey was administered in Japanese and used a 2-part branching structure. On the landing page, respondents received a brief explanation of the survey and the optional section. Those who chose to continue to Part 2 then saw a second brief explanation stating that the following items asked for more detailed opinions about men’s health, including reproductive function. Part 1 was mandatory and assessed awareness of the parent study, reasons for nonparticipation (multiple responses allowed), interest in information on male reproductive ability, and general openness toward future semen analysis or similar research. Part 2 was optional and assessed age, awareness of the association between sperm condition and overall health, perceived importance of male health for future conception, self-reported resistance to undergoing semen analysis, anxiety about poor results, concerns about collection location or privacy protection, expected reactions of others, and willingness to undergo semen analysis under simplified conditions. Specifically, this final item assessed hypothetical willingness to undergo semen analysis if cost, location, and collection method were easier; it should not be interpreted as willingness to participate in a specific future research protocol. Although the overall study was framed as a formative follow-up survey of nonparticipation in the parent study, several of these Part 2 items assessed broader reproductive health knowledge and affective responses to semen analysis. These items should therefore be interpreted as exploratory ancillary responses collected under the implemented survey framing, not as validated measures of general male reproductive health knowledge, underlying affective constructs, or population-level acceptability. The resistance item was treated as a single study-specific self-report item and not as a validated measure of a psychological construct.

The complete original Japanese questionnaire, provided as a PDF export of the Google Form used for data collection, is included as Multimedia Appendix 2. An English translation of all items and response options is provided as Multimedia Appendix 3. No personal identifiers were collected, and IP addresses and platform server logs were not accessed. Required response settings were used for the structured items displayed to respondents within each selected section. Part 2 was optional, and the final free-text field was optional. Respondents could review and change their answers before submission. No item randomization was used. Because technical prevention of duplicate entries was not feasible, the dataset was screened manually for obvious duplicates, such as identical response patterns with the same timestamp.

Because the optional section was introduced broadly as asking about men’s health, including reproductive function, whereas several analyzed items assessed affective or procedural concerns, variables in this paper are labeled according to the actual item content. Furthermore, because several participant-facing terms were broad, variable labels follow the most conservative interpretation of each item. In particular, the item using “cooperate with” future semen analysis or similar research is described as general openness rather than willingness to participate in a specific future protocol. Similarly, reasons for nonparticipation are interpreted as reported reasons under the original item wording, which referred to semen analysis as the most salient component of the parent study. Possible response framing from this mismatch and the specific phrasing of items is addressed as a limitation.

### Statistical Analysis

Participant characteristics and response distributions were summarized descriptively, and percentages were calculated. To account for the sample size, 95% CIs for proportions were calculated using the Wilson score interval method [45]. Given the limited sample size, response categories were aggregated where appropriate. For 5-point Likert scale items, the top 2 categories (eg, “Strongly” and “To some extent”) were combined to indicate the presence of barriers, and other categorical responses were grouped on the basis of conceptual relevance [46]. All analyses of Part 2 variables were restricted to respondents who completed the optional section. Because only 6 employees completed the parent semen-analysis protocol, direct comparisons between participants and nonparticipants were not feasible. No formal hypothesis testing was planned, and findings were interpreted descriptively.

The main descriptive analysis distinguished respondents who self-reported no prior awareness of the parent study from those who reported at least some prior awareness. This classification was treated as an indicator of effective recruitment reach, not as objective evidence of exposure or nonexposure to recruitment materials. Respondents were classified as “unaware” if they selected that they had not known the study was being conducted and as “aware nonparticipants” if they reported any prior awareness but had not completed the parent protocol. We did not label aware respondents as “informed decliners” because the survey did not assess comprehension of the occupational rationale or the details of the parent study. Because multiple responses were allowed for reasons for nonparticipation, proportions were recalculated using stratum-specific denominators.

As secondary descriptive sensitivity analyses, we further divided the aware stratum into respondents who were aware but poorly informed and those who knew some of the content or had considered participating. We also explored Part 2 responses by awareness status among Part 2 completers. These subgroup analyses were interpreted as supportive descriptive context rather than confirmatory evidence. A small number of internally inconsistent responses were retained to preserve the integrity of the anonymous self-administered dataset and were interpreted cautiously.

All statistical analyses were performed using R statistical software (version 4.3.1; R Foundation for Statistical Computing). Responses to the optional free-text field were reviewed for contextual understanding but were not analyzed qualitatively because systematic qualitative methods had not been prespecified.

## Results

### Survey Participation, Completion, and Data Quality

Data screening identified no obvious duplicate entries, and no missing values were present. Approximately 900 male employees were invited to the survey, and 108 analyzable submitted questionnaires were obtained. Because the exact numbers of employees who received, viewed, or started the survey were unavailable, no formal response rate was reported. Of the 108 respondents, 83 completed the optional detailed section (Part 2), yielding a Part 2 completion proportion of 76.9%.

### Part 1 (Mandatory): Awareness, Reported Reasons, Information Needs, and General Openness

The key descriptive finding was that nonparticipation was heterogeneous. Most respondents had not been aware of the parent study, whereas the smaller group with prior awareness reported a different pattern of reasons centered on perceived irrelevance, semen-collection resistance, embarrassment or reluctance, and hassle.

Table 1 summarizes the overall Part 1 responses. Of the 108 respondents, 74 (68.5%; 95% CI 59.3‐76.5) were classified as unaware of the parent study and 34 (31.5%; 95% CI 23.5‐40.7) as aware nonparticipants. No respondent selected the option indicating actual participation in the parent study, confirming that the survey targeted nonparticipants. Interest in information on male reproductive ability was limited: 30/108 (27.8%; 95% CI 20.2‐36.9) respondents answered “Yes,” 49/108 (45.4%; 95% CI 36.3‐54.8) answered “Neither,” and 29/108 (26.9%; 95% CI 19.4%‐35.9%) answered “No.” Regarding general openness toward future semen analysis or similar research, 11/108 (10.2%; 95% CI 5.8‐17.3) respondents indicated that they would like to cooperate, and 44/108 (40.7%; 95% CI 31.9‐50.2) indicated that they would cooperate if conditions were met (Table 1). Because this item used the broad wording “cooperate with” and did not describe a specific future study protocol, it should be interpreted only as a measure of general openness to related future research.

**Table 1.** Overall Part 1 responses: awareness of the parent study, interest in information on male reproductive ability, and general openness toward future-related research (N=108).

Question and response category	Respondents, n (%)^a^	95% CI^b^
Awareness of the parent study		
Unaware	74 (68.5)	59.3‐76.5
Aware nonparticipants^c^	34 (31.5)	23.5‐40.7
Interest in information on male reproductive ability		
Yes	30 (27.8)	20.2‐36.9
Neither	49 (45.4)	36.3‐54.8
No	29 (26.9)	19.4‐35.9
General openness toward future semen analysis or similar research		
Want to cooperate	11 (10.2)	5.8‐17.3
Would cooperate if conditions are met	44 (40.7)	31.9‐50.2
Do not know	36 (33.3)	25.2‐42.7
Do not intend to cooperate	17 (15.7)	10.1‐23.8

a Values are presented as n (% of respondents), unless otherwise specified. Percentages were calculated using the total number of respondents to Part 1 of the questionnaire (N=108) as the denominator.

b CIs for proportions were calculated using the Wilson score interval method.

c “Aware nonparticipants” includes respondents who reported some prior awareness of the parent study in Question 1 but did not participate. No respondent selected the option indicating actual participation in the parent study.

### Stratified Analysis of Reasons for Nonparticipation

Table 2 presents the Q1-based stratified analysis of reasons for nonparticipation. Within the unaware group, 68/74 (91.9%; 95% CI 83.4‐96.2) respondents selected “Did not know the study existed” as a reason for nonparticipation. Only 6/74 (8.1%; 95% CI 3.8‐16.6) respondents did not endorse this option; among these internally inconsistent responses, alternative reasons were infrequent, with “Felt it was a hassle” the most common (2/74, 2.7%).

Among aware nonparticipants, the most frequently endorsed reasons were perceived irrelevance (9/34, 26.5%; 95% CI 14.6‐43.1), resistance to collecting semen (9/34, 26.5%; 95% CI 14.6‐43.1), embarrassment or reluctance (8/34, 23.5%; 95% CI 12.4‐40), and feeling that participation would be a hassle (7/34, 20.6%; 95% CI 10.3‐36.8). Other reasons included insufficient understanding of the participation method or study content (5/34, 14.7%), being too busy (5/34, 14.7%), concerns about the collection location or method (4/34, 11.8%), and time or location mismatch (4/34, 11.8%). Overall, after stratification by study awareness, lack of awareness predominated among respondents reporting no prior knowledge of the study, whereas the barrier profile among aware nonparticipants centered on low perceived relevance, embarrassment, and procedural or procedure-related burden (Table 2).

**Table 2.** Awareness-stratified reasons for not undergoing the semen analysis component of the parent study (Question 2; multiple responses allowed; N=108).

Reason for nonparticipation^a^	Overall, n/N (%; 95% CI)^b^	Unaware^c^, n/N (%; 95% CI^b^	Aware nonparticipants^d^, n/N (%; 95% CI)^b^
Did not know the study existed	68/108 (63; 53.6‐71.5)	68/74 (91.9; 83.4‐96.2)	0/34 (0; 0‐10.2)
Perceived as irrelevant	10/108 (9.3; 5.1‐16.2)	1/74 (1.4; 0.2‐7.3)	9/34 (26.5; 14.6‐43.1)
Knew about the study but felt embarrassed or reluctant	9/108 (8.3; 4.4‐15.1)	1/74 (1.4; 0.2‐7.3)	8/34 (23.5; 12.4‐40.0)
Resistance to collecting semen itself	9/108 (8.3; 4.4‐15.1)	0/74 (0; 0‐4.9)	9/34 (26.5; 14.6‐43.1)
Felt it was a hassle	9/108 (8.3; 4.4‐15.1)	2/74 (2.7; 0.7‐9.3)	7/34 (20.6; 10.3‐36.8)
Did not understand the participation method or study content well	7/108 (6.5; 3.2‐12.8)	2/74 (2.7; 0.7‐9.3]	5/34 (14.7; 6.4‐30.1)
Too busy to make time	6/108 (5.6; 2.6‐11.6)	1/74 (1.4; 0.2‐7.3)	5/34 (14.7; 6.4‐30.1)
Concerned about the collection location or method	5/108 (4.6; 2.0‐10.4)	1/74 (1.4; 0.2‐7.3)	4/34 (11.8; 4.7‐26.6)
Time or location did not work	5/108 (4.6; 2.0‐10.4)	1/74 (1.4; 0.2‐7.3]	4/34 (11.8; 4.7‐26.6)
Did not feel it was necessary because I do not want children in the future	4/108 (3.7; 1.4‐9.1)	1/74 (1.4; 0.2‐7.3)	3/34 (8.8; 3‐23)
Was not motivated because others were not participating	4/108 (3.7; 1.4‐9.1)	1/74 (1.4; 0.2‐7.3)	3/34 (8.8; 3‐23)
Worried that people at my workplace would find out	3/108 (2.8; 0.9‐7.9)	0/74 (0; 0‐4.9)	3/34 (8.8; 3‐23.0)
Other (free text)^e^^,^^f^	3/108 (2.8; 0.9‐7.9)	2/74 (2.7; 0.7‐9.3)	1/34 (2.9; 0.5‐14.9)
Missed the procedures or timing	2/108 (1.9; 0.5‐6.5)	1/74 (1.4; 0.2‐7.3)	1/34 (2.9; 0.5‐14.9)
Did not want family or partner to know	1/108 (0.9; 0.2‐5.1)	1/74 (1.4; 0.2‐7.3)	0/34 (0; 0‐10.2)
Afraid of knowing the test results	0/108 (0; 0‐3.4)	0/74 (0; 0‐4.9)	0/34 (0; 0‐10.2)

a Because Question 2 allowed multiple responses, percentages may sum to more than 100%.

b Values are presented as n/N (% within the group), with 95% CIs calculated using the Wilson score method.

c “Unaware” was defined as respondents who selected “I was not aware that the study was being conducted” in Question 1.

d “Aware nonparticipants” was defined as respondents who reported any prior awareness in Question 1 but did not participate in the parent study.

e Example free-text responses in the pooled sample were “Thought I was not eligible,” “Because I already have children,” and “Because I have already undergone postvasectomy” (1 response each).

f A small number of respondents classified as “Unaware” in Question 1 did not endorse “Did not know the study existed” in Question 2. These internally inconsistent responses were retained to preserve the integrity of the anonymous self-administered dataset and were interpreted cautiously.

### Part 2 (Optional): Exploratory Ancillary Responses on Reproductive Health Knowledge, Concerns, and Willingness Under Simplified Conditions

Table 3 summarizes the optional Part 2 responses among 83 completers. Most respondents were aged 40 years or older (56/83, 67.5%; 95% CI 56.8‐76.6). Knowledge of the association between sperm condition and overall health was limited: only 3/83 (3.6%; 95% CI 1.2‐10.1) respondents answered that they knew this well, whereas 48/83 (57.8%; 95% CI 47.1‐67.9) answered that they did not know it. In contrast, 79/83 (95.2%; 95% CI 88.3‐98.1) respondents considered male health important for future conception. Self-reported concerns about semen analysis were common in this subgroup. A single study-specific item on resistance to undergoing semen analysis was endorsed by 42/83 (50.6%; 95% CI 40.1‐61.1) respondents, anxiety about potentially poor results by 52/83 (62.7%; 95% CI 51.9‐72.3), and concerns about collection location or privacy protection by 48/83 (57.8%; 95% CI 47.1‐67.9). Expected resistance from others was less common, with 27/83 (32.5%; 95% CI 23.4‐43.2) respondents anticipating strong or slight resistance from family, partners, or friends. Under simplified conditions, 36/83 (43.4%; 95% CI 33.2‐54.1) respondents answered that they would undergo semen analysis, 33/83 (39.8%; 95% CI 29.9‐50.5) answered “Do not know,” and 14/83 (16.9%; 95% CI 10.3‐26.3) answered “No” (Table 3).

**Table 3.** Overall Part 2 responses: respondent characteristics, knowledge, reported concerns, and willingness under simplified conditions.

Question and response category^a^	Respondents, n (%)^b^	95% CI^c^
Age (years)		
29 or younger	5 (6)	2.6‐13.3
30‐39	22 (26.5)	18.2‐36.9
40 or older (subtotal)^d^	56 (67.5)	56.8‐76.6
40‐49	27 (32.5)	23.4‐43.2
50 or older	29 (34.9)	25.6‐45.7
Awareness of the association between sperm condition and systemic health		
Know well	3 (3.6)	1.2‐10.1
Heard of it	32 (38.6)	28.8‐49.3
Do not know	48 (57.8)	47.1‐67.9
Perception of the importance of male health for future conception		
Important (very + to some extent)^e^	79 (95.2)	88.3‐98.1
Think it is very important	53 (63.9)	53.1‐73.4
Think it is important to some extent	26 (31.3)	22.4‐41.9
Neither	3 (3.6)	1.2‐10.1
Do not think it is very important	1 (1.2)	0.2‐6.5
Do not think it is important at all	0 (0)	0‐4.4
Self-reported resistance to undergoing semen analysis^f^		
Feel resistance (strongly+ to some extent)^e^	42 (50.6)	40.1‐61.1
Feel very strongly	7 (8.4)	4.1‐16.4
Feel to some extent	35 (42.2)	32.1‐52.9
Neither	20 (24.1)	16.2‐34.3
Do not feel much	13 (15.7)	9.4‐25
Do not feel at all	8 (9.6)	5‐17.9
Anxiety about potential poor semen analysis results^f^		
Feel anxiety (strongly+ to some extent)^e^	52 (62.7)	51.9‐72.3
Feel very strongly	20 (24.1)	16.2‐34.3
Feel to some extent	32 (38.6)	28.8‐49.3
Neither	17 (20.5)	13.2-30.4
Do not feel much	8 (9.6)	5.0‐17.9
Do not feel at all	6 (7.2)	3.4‐14.9
Concerns about the collection location or privacy protection^f^		
Feel concerns (strongly+ to some extent)^e^	48 (57.8)	47.1‐67.9
Feel very strongly	17 (20.5)	13.2‐30.4
Feel to some extent	31 (37.3)	27.7‐48.1
Neither	14 (16.9)	10.3‐26.3
Do not feel much	16 (19.3)	12.2‐29
Do not feel at all	5 (6)	2.6‐13.3
Expected reaction of others (family, partner, friends, etc) to semen analysis		
Expect resistance (Strong+ Slight)^e^	27 (32.5)	23.4‐43.2
Expect strong resistance	6 (7.2)	3.4‐14.9
Expect slight resistance	21 (25.3)	17.2‐35.6
Expect they will feel nothing	25 (30.1)	21.3‐40.7
Expect they will be supportive	17 (20.5)	13.2‐30.4
Do not know	14 (16.9)	10.3‐26.3
Willingness to undergo semen analysis under simplified conditions		
Yes	36 (43.4)	33.2‐54.1
No	14 (16.9)	10.3‐26.3
Do not know	33 (39.8)	29.9‐50.5

a Part 2 was optional; therefore, these estimates describe a self-selected subgroup and should not be interpreted as prevalence estimates for all invited workers or all nonparticipants.

b Values are presented as n (% of Part 2 completers), unless otherwise specified. Percentages were calculated using the total number of respondents to Part 2 of the questionnaire (n=83) as the denominator.

c CIs for proportions were calculated using the Wilson score interval method.

d Subcategories under each main response category are shown for descriptive purposes and are included in the corresponding subtotal.

e Composite categories were created by aggregating adjacent response options on the Likert scale to facilitate interpretation.

f The resistance, anxiety, and collection-location or privacy concern items were study-specific self-report items and should not be interpreted as validated psychometric measures.

Table 4 presents a secondary descriptive sensitivity analysis in which the aware stratum was divided into respondents who knew that the study was being conducted but did not understand its content well (n=17) and those who knew some of the content or had considered participating (n=17). In the former subgroup, embarrassment or reluctance and resistance to collecting semen were each reported by 5/17 (29.4%) respondents, followed by perceived irrelevance and insufficient understanding of the participation method or study content (each 4/17, 23.5%). In the latter subgroup, perceived irrelevance was most common (5/17, 29.4%), followed by resistance to collecting semen and feeling that participation would be a hassle (each 4/17, 23.5%), and being too busy or experiencing a time or location mismatch (each 3/17, 17.6%). These patterns were consistent with the primary 2-strata analysis and are presented as supportive context rather than formal subgroup testing (Table 4).

**Table 4.** Secondary descriptive sensitivity analysis of reasons for nonparticipation using 3 awareness strata (Question 2; multiple responses allowed).

Reason for nonparticipation^a^^,b^	Unaware^c^, n/N (%; 95% CI)^d^	Aware but poorly informed^e^, n/N (%; 95% CI)^d^	Aware nonparticipants with greater study knowledge or engagement^f^, n/N (%; 95% CI)^d^
Did not know the study existed	68/74 (91.9; 83.4‐96.2)	0/17 (0; 0‐18.4)	0/17 (0; 0‐18.4)
Perceived as irrelevant	1/74 (1.4; 0.2‐7.3)	4/17 (23.5; 9.6‐47.3)	5/17 (29.4; 13.3‐53.1)
Knew about the study but felt embarrassed or reluctant	1/74 (1.4; 0.2‐7.3)	5/17 (29.4; 13.3‐53.1)	3/17 (17.6; 6.2‐41)
Resistance to collecting semen itself	0/74 (0; 0‐4.9)	5/17 (29.4; 13.3‐53.1)	4/17 (23.5; 9.6‐47.3)
Felt it was a hassle	2/74 (2.7; 0.7‐9.3)	3/17 (17.6; 6.2‐41)	4/17 (23.5; 9.6‐47.3)
Did not understand the participation method or study content well	2/74 (2.7; 0.7‐9.3)	4/17 (23.5; 9.6‐47.3)	1/17 (5.9; 1‐27)
Too busy to make time	1/74 (1.4; 0.2‐7.3)	2/17 (11.8; 3.3‐34.3)	3/17 (17.6; 6.2‐41)
Concerned about the collection location or method	1/74 (1.4; 0.2‐7.3)	2/17 (11.8; 3.3‐34.3)	2/17 (11.8; 3.3‐34.3)
Time or location did not work	1/74 (1.4; 0.2‐7.3)	1/17 (5.9; 1‐27)	3/17 (17.6; 6.2‐41)
Did not feel it was necessary because I do not want children in the future	1/74 (1.4; 0.2‐7.3)	3/17 (17.6; 6.2‐41)	0/17 (0; 0‐18.4)
Was not motivated because others were not participating	1/74 (1.4; 0.2‐7.3)	2/17 (11.8; 3.3‐34.3)	1/17 (5.9; 1‐27)
Worried that people at my workplace would find out	0/74 (0; 0‐4.9)	2/17 (11.8; 3.3‐34.3)	1/17 (5.9; 1‐27)
Other (free text)	2/74 (2.7; 0.7‐9.3)	0/17 (0; 0‐18.4)	1/17 (5.9; 1‐27)
Missed the procedures or timing	1/74 (1.4; 0.2‐7.3)	1/17 (5.9; 1‐27)	0/17 (0; 0‐18.4)
Did not want family or partner to know	1/74 (1.4; 0.2‐7.3)	0/17 (0; 0‐18.4)	0/17 (0; 0‐18.4)
Afraid of knowing the test results	0/74 (0; 0‐4.9)	0/17 (0; 0‐18.4)	0/17 (0; 0‐18.4)

a Because Question 2 allowed multiple responses, percentages may sum to more than 100%.

b This was a secondary descriptive sensitivity analysis and was not prespecified. It should be interpreted as supportive context for the primary 2-strata analysis rather than as formal subgroup testing.

c “Unaware” was defined as respondents who selected “I was not aware that the study was being conducted” in Question 1.

d Values are presented as n/N (% within the group), with 95% CIs calculated using the Wilson score method.

e “Aware but poorly informed” was defined as respondents who selected “I knew the study was being conducted, but I did not really understand the details” in Question 1.

f “Aware nonparticipants with greater study knowledge or engagement” was defined as respondents who selected either “I knew the study details to some extent, but I did not participate” or “I considered participating but ultimately did not participate” in Question 1.

Exploratory awareness-stratified analyses of optional Part 2 items are shown in Table 5. Among Part 2 completers, self-reported resistance to undergoing semen analysis was endorsed more frequently by aware completers than by unaware completers (20/30, 66.7% vs 22/53, 41.5%), whereas anxiety about poor results (17/30, 56.7% vs 35/53, 66%) and concerns about collection location or privacy protection (17/30, 56.7% vs 31/53, 58.5%) were similar between groups. Willingness to undergo semen analysis under simplified conditions was 50% (15/30) among aware completers and 39.6% (21/53) among unaware completers. Because Part 2 was optional and completion differed by awareness status, these comparisons should be interpreted cautiously.

**Table 5.** Exploratory awareness-stratified analysis of optional Part 2 items among completers.

Item^a^	Overall, n/N (%; 95% CI)^b^	Aware completers^c^^,^^d^ (n=30), n/N (%; 95% CI)^b^	Unaware completers^c^^,^^d^ (n=53), n/N (%; 95% CI)^b^
Male health knowledge (know well + heard of it)^e^	35/83 (42.2; 32.1‐52.9)	10/30 (33.3; 19.2‐51.2)	25/53 (47.2; 34.4‐60.3)
Male health important (very + to some extent)^f^	79/83 (95.2; 88.3‐98.1)	29/30 (96.7; 83.3‐99.4)	50/53 (94.3; 84.6‐98.1)
Self-reported resistance to semen analysis (very + to some extent)^g^	42/83 (50.6; 40.1‐61.1)	20/30 (66.7; 48.8‐80.8)	22/53 (41.5; 29.3‐54.9)
Anxiety about poor results (very + to some extent)^g^	52/83 (62.7; 51.9‐72.3)	17/30 (56.7; 39.2‐72.6)	35/53 (66; 52.6‐77.3)
Concerns about collection location or privacy protection (very + to some extent)^g^	48/83 (57.8; 47.1‐67.9)	17/30 (56.7; 39.2‐72.6)	31/53 (58.5; 45.1‐70.7)
Expected resistance from others (strong + slight)^h^	27/83 (32.5; 23.4‐43.2)	10/30 (33.3; 19.2‐51.2)	17/53 (32.1; 21.1‐45.5)
Would undergo semen analysis under simplified conditions	36/83 (43.4; 33.2‐54.1)	15/30 (50; 33.2‐66.8)	21/53 (39.6; 27.6‐53.1)

a This was an exploratory analysis of an optional survey section and should not be interpreted as confirmatory subgroup evidence.

b Values are presented as n/N (% within the group), with 95% CIs calculated using the Wilson score method.

c Awareness status was defined using Question 1 among all Part 1 respondents included in the analysis; this table includes only respondents who completed optional Part 2.

d Part 2 completion differed by awareness status (aware: 30/34, 88.2%; unaware: 53/74, 71.6%); therefore, these comparisons are descriptive and should be interpreted cautiously.

e “Male health knowledge” combines “I know this well” and “I have heard something about it.”

f “Male health important” combines “very important” and “important to some extent.”

g “Self-reported resistance to undergoing semen analysis,” “Anxiety about poor results,” and “Concerns about collection location or privacy protection” combine the top 2 Likert categories. These were study-specific self-report items and should not be interpreted as validated psychometric scales.

h “Expected resistance from others” combines “strong resistance” and “slight resistance.”

## Discussion

### Principal Findings

The main contribution of this study is not the observation that workers who were unaware of a study did not participate, but the identification of a denominator problem in interpreting very low uptake after a sensitive employer-adjacent biospecimen recruitment attempt. The very low uptake in the parent workplace semen analysis study could not be interpreted as a single endpoint or as simple refusal of semen analysis. In this follow-up sample, most respondents reported no prior awareness of the parent study, whereas respondents with prior awareness reported a different profile of reasons, including low perceived relevance, resistance to collecting semen, embarrassment or reluctance, and hassle. Without separating recruitment reach from nonparticipation after prior awareness, low uptake may be incorrectly attributed to population-level acceptability issues, disinterest, or psychological resistance. This interpretation is consistent with process-evaluation guidance, which emphasizes reach, implementation, mechanisms, and context [47]. It is also consistent with recruitment-failure literature showing that poor enrollment may reflect multiple interacting design, communication, setting, and participant-level factors rather than a single participant-level cause [4,48].

This finding should be interpreted as a limited post hoc process evaluation. We did not prospectively measure objective exposure to recruitment materials, whether the materials were opened or read, whether the occupational reproductive health rationale was understood, or whether nonparticipation represented informed declination. Therefore, the findings do not show that lack of awareness, low perceived relevance, or semen-collection concerns were the primary causal determinants of nonparticipation. In asymptomatic workplace research, declining semen analysis is a legitimate choice and should be respected [26]. In employer-adjacent occupational research, privacy, nondiscrimination, and power imbalance require particular attention [27]. Future studies should therefore instrument the recruitment pathway more carefully before interpreting low uptake, including objective exposure to recruitment materials, access to study information, understanding of the occupational rationale, voluntary postinformation declination, privacy concerns, logistical burden, and remaining procedure-related concerns.

### Comparison With Prior Work and Interpretation

The procedure-related concerns reported by aware nonparticipants are consistent with prior work showing that semen testing may be associated with embarrassment, stigma, anxiety, and reluctance to participate [7,8]. The self-reported resistance item is therefore treated only as a descriptive response to a study-specific survey item, not as evidence of a new or validated psychological construct. The added value of this study is contextual and methodological: the survey was conducted after an actual workplace semen analysis recruitment attempt with extremely low uptake, and the awareness-stratified analysis separated respondents who reported no prior awareness of the study from those who reported some awareness of the study.

The discrepancy regarding anxiety between Part 1 and Part 2 should be interpreted cautiously. Fear of poor results was uncommon as a retrospective reason for nonparticipation in Part 1, whereas result-related anxiety was common among Part 2 completers. This pattern may reflect differences in item framing, the optional and self-selected nature of Part 2, and a preference for more socially acceptable explanations when describing a past decision [44,49]. This difference should therefore be treated as hypothesis-generating and should not be interpreted as evidence that result-related anxiety explained prior nonparticipation.

Nevertheless, although not directly comparable, the level of result-related anxiety observed in the optional subgroup was within the broad range reported before several other medical procedures, suggesting that this concern should not be dismissed as trivial [50-52]. In an asymptomatic workplace setting, this supports the need for careful consent processes and a meaningful choice about whether to receive individual results, including respect for the right not to know [30,32]. While prior work has shown substantial heterogeneity in how risks and benefits are communicated in consent materials and that understanding of core consent elements is often incomplete [30,31], we did not assess consent comprehension in either the parent study or the present survey. These references are thus cited primarily as general ethical context for future workplace recruitment strategies.

### What This Study Does and Does Not Show

This study does not show that workers made an informed judgment that semen testing was irrelevant to them. The follow-up survey did not include occupational reproductive-hazard education, did not assess comprehension of the exposure-related rationale, and did not evaluate willingness after balanced contextual information. Low perceived relevance may therefore reflect the implemented information condition rather than a stable attitude toward occupational reproductive-health research. Prior research suggests that men’s knowledge of fertility and preconception health is often limited, and occupational reproductive hazards may not be salient to asymptomatic workers without contextual explanation [18,23,33]. This limitation is important, and future studies should provide neutral, balanced information about the occupational and environmental rationale for semen testing, assess comprehension, and then document voluntary post-information declination or consent [53].

The study also does not establish new psychological constructs related to semen collection. The resistance, anxiety, and privacy-related items were study-specific self-report items. Their value is formative: they point to design elements that should be addressed and measured prospectively, including collection location, specimen transport, privacy protections, return-of-results preferences, and the separation of research data from employers [27,54-57].

### Practical Implications

Importantly, this study does not advocate for the broad implementation of routine workplace semen-based assessments as a cost-effective occupational health intervention. Rather, such assessments should be considered only when there is a clear exposure-defined research question and strong privacy and voluntariness protections are in place. Accordingly, future exposure-defined workplace reproductive-health studies should treat recruitment as a cascade rather than as a single uptake proportion. At minimum, future protocols should prospectively document: (1) the number of eligible employees, (2) the number objectively exposed to recruitment materials, (3) the number who accessed participant information, (4) the number who demonstrated understanding of the occupational reproductive health rationale, (5) the number who declined after adequate information, (6) structured reasons for informed declination, (7) privacy and logistical concerns, (8) consent, (9) collection attempt, (10) specimen return, and (11) usable specimen completion. This staged approach is consistent with process-evaluation guidance and recruitment-failure literature emphasizing that reach, implementation, context, and participant response should be distinguished when interpreting low enrollment.

These stages have different ethical and practical meanings. Limited reach calls for improved communication channels. Insufficient understanding calls for clearer and more contextualized participant information. Informed declination should be respected as an appropriate exercise of autonomy. Procedure-related concerns call for the redesign of collection, transport, privacy protection, and result-return procedures. Because semen collection has distinctive privacy and logistical burdens, future occupational reproductive health studies should prospectively compare alternative collection pathways, including more private or home-based approaches where analytically appropriate. Collection setting is not merely a logistical detail; semen collection at home versus at the clinic has been empirically studied and may affect both acceptability and measured semen parameters.

To address these procedure-related concerns more concretely, future studies may evaluate candidate design elements such as more private home-based testing options [58]. Additionally, emerging adjunctive approaches based on serum hormones or clinical, endocrine, and testicular factors may help triage or contextualize male reproductive assessment [59,60], although these should not be treated as immediate replacements for semen analysis. Ultimately, standardized multimodal databases, such as the recently initiated N-SEED (Nippon Semen and Environmental Exposure Database), may support the future development and validation of image-based and exposure-integrated fertility assessment methods, which could eventually inform the development of less burdensome study designs [61].

### Limitations

This study has some limitations. First, because it included 108 respondents from a single-company convenience sample of nonparticipants, the findings should be interpreted as formative and preliminary rather than as population estimates. Selection bias is likely, as respondents may have differed from nonrespondents in health interest, reproductive-health concerns, or willingness to answer a sensitive survey. We do not claim statistical generalizability to workplace surveys in general. The specific barrier frequencies reported here are most relevant to workplace-based reproductive health research involving semen collection or similarly sensitive biological procedures. The more transferable implication is methodological: very low uptake should not be interpreted as a single phenomenon, and future studies should distinguish objectively measured nonexposure to recruitment materials—or, when only survey data are available, self-reported unawareness—from declination after prior awareness before attributing nonparticipation to population-level acceptability, perceived relevance, or reported affective and procedure-related barriers.

Second, because invitations were distributed offline and no prospective analytics or log tracking was used, the exact denominator, view rate, and start rate were unavailable. Consequently, our measurement of study awareness relied entirely on self-report, which is susceptible to recall bias, inattention, or a failure to notice recruitment materials. We could not objectively verify whether informational materials physically reached the respondents. This limitation applies to recruitment-reach metrics only; all respondent-level analyses used exact counts from submitted questionnaires. Future studies should prospectively use a separate tracked landing page or an equivalent method, if ethically acceptable, when exact reach metrics are required.

Third, Part 2 was optional. Respondents who proceeded to it may have been more aware of the parent study, more interested in male reproductive health, less time-constrained, or more motivated to express concerns. Consistent with this possibility, Part 2 completion was higher among aware than unaware respondents (30/34, 88.2% vs 53/74, 71.6%). The Part 2 findings should therefore be interpreted as descriptive of a self-selected subgroup rather than as prevalence estimates for all nonparticipants.

Fourth, the questionnaire used study-specific items with expert face review but no formal pilot testing or cognitive interviewing. Some participant-facing wording was simplified and not fully specific; in particular, the landing-page and optional-section descriptions were broad and did not explicitly enumerate all analyzed item domains, including self-reported resistance, anxiety, collection location, and privacy protection. One item also combined collection location and privacy protection. In addition, the use of “semen analysis” as shorthand for the entire research project and “cooperate with” instead of “participate in” may have introduced framing bias [62]. These wording limitations reduce construct precision and support the conservative interpretation adopted in this paper.

A related reporting and interpretation issue concerns the relationship between the participant-facing explanation and the inferential framing of the present report. The implemented survey conditions support only a restricted interpretation: the data can describe self-reported awareness, reported reasons for nonparticipation, and study-specific concerns under the implemented information condition, but they cannot support claims about causal determinants, population-level acceptability, or informed attitudes toward occupational reproductive-health research. This restricted framing represents a conservative alignment of the anonymous dataset with the implemented survey and consent conditions, rather than a retrospective expansion of the study objective.

Finally, a key limitation is the information condition under which responses were collected. The follow-up survey did not include an occupational reproductive-hazard education module, did not assess comprehension of the exposure-related rationale for the parent study, and did not measure willingness after such information. Therefore, the data cannot determine whether low perceived relevance represented an informed judgment or reflected insufficient occupational-health contextualization. Low perceived relevance should thus be interpreted as a response under the implemented information condition, not as evidence that semen testing would continue to be perceived as irrelevant after balanced occupational reproductive-health explanation. A future study designed to answer that question would need to provide neutral and balanced occupational reproductive-health information, assess comprehension, and then measure voluntary postinformation consent or declination.

### Conclusions

In this post hoc formative process evaluation, very low uptake in a workplace semen analysis study reflected multiple points in the recruitment cascade rather than a single phenomenon. Most respondents reported no prior awareness of the parent study, whereas aware nonparticipants reported low perceived relevance and semen collection–related concerns. The findings support a bounded recruitment-methodological interpretation rather than a causal explanation for nonparticipation or an estimate of informed acceptability after occupational reproductive-hazard education. Future exposure-defined workplace reproductive health studies should prospectively measure recruitment reach, information access, comprehension of the occupational rationale, voluntary postinformation declination, privacy concerns, logistical burden, and specimen completion as separate stages of a recruitment cascade.

## Supplementary material

10.2196/90788 Multimedia Appendix 1 Summary of the originally planned workplace-based epidemiological project, including study design, objectives, target population, recruitment methods, and data and sample collection procedures.

10.2196/90788 Multimedia Appendix 2 Original Japanese version of the web-based questionnaire as administered to participants, provided as a PDF export of the Google Form used for data collection.

10.2196/90788 Multimedia Appendix 3 English translation of the questionnaire items and response options, provided to ensure transparency and reproducibility for non–Japanese-speaking readers.

10.2196/90788 Checklist 1 CHERRIES checklist.

10.2196/90788 Checklist 2 CROSS checklist.

## Abbreviations

CHERRIES: Checklist for Reporting Results of Internet E-Surveys
CROSS: Consensus-Based Checklist for Reporting of Survey Studies
N-SEED: Nippon Semen and Environmental Exposure Database
