# Supplementary material for: Very Low Uptake in Workplace Semen Analysis Research: Formative Web-Based Cross-Sectional Follow-Up Survey Distinguishing Employees With Self-Reported Unawareness From Aware Nonparticipants
Source: JMIR Form Res. 2026 Jul 13;10:e90788. doi: 10.2196/90788 (PMC13361622; doi:10.2196/90788)
Supplement: Multimedia Appendix 1 [file formative-v10-e90788-s001.docx]

**Multimedia Appendix 1**

**Table S1.** Outline of the Originally Planned Epidemiological Project.

| Item | Description |
| --- | --- |
| Study Design | Cross-sectional study involving questionnaire survey and biological sample analysis |
| Objective | To clarify the effects of environmental exposure and three-shift work on fertility in working-age men |
| Primary Outcomes | Semen parameters (semen volume, sperm concentration, motility, and morphology) related to work schedule, lifestyle habits, and environmental factors |
| Secondary Outcomes | Urinalysis (organic solvent metabolites, specific gravity, creatinine) |
| Target Population | Approximately 2,000 male employees at three cooperating companies with three-shift systems in the medical care region of the International University of Health and Welfare Hospital (Nasushiobara, Japan) |
| Target Sample Size | Minimum of 30 participants (established based on feasibility for an exploratory pilot analysis without formal power calculations, while aiming to recruit as many as possible) |
| Inclusion Criteria | - Age: 18–65 years  - Employment: Clear work schedule (day shift or three-shift)  - Consent: Obtained for research participation |
| Exclusion Criteria | - History of genital disease  - Past indication of chromosomal abnormalities |
| Recruitment Period | November 2024 to January 2025 |
| Recruitment Methods | Flyers and digital signage with QR codes (DENSO WAVE Incorporated) linked to the application form |
| Incentives | Free semen analysis (valued at 5,000 JPY) |
| Data & Sample Collection | - Data: Collected via Google Form (Mountain View, CA, USA)  - Semen Analysis: Participants received a collection container by mail and submitted the collected specimen to the laboratory in person |
