# Supplementary material for: Very Low Uptake in Workplace Semen Analysis Research: Formative Web-Based Cross-Sectional Follow-Up Survey Distinguishing Employees With Self-Reported Unawareness From Aware Nonparticipants
Source: JMIR Form Res. 2026 Jul 13;10:e90788. doi: 10.2196/90788 (PMC13361622; doi:10.2196/90788)
Supplement: Multimedia Appendix 2 [file formative-v10-e90788-s002.pdf]

# 製造業に従事する男性を対象とした、健康についてのアンケート

このアンケートは、製造業に従事する男性を対象に2024年11月〜2025年1月に実施された「男性の健康（アンケート・精液検査・尿検査）に関する研究」への参加状況や、ご自身の健康に対する考え方についてお伺いするものです。

● 回答はすべて匿名・自由意志です（2〜3分程度で終了します）
● いただいた回答は、研究データの分析や今後の調査方法の改善などを目的として利用させていただきます
●アンケートへの回答を以て同意を得たものとします
● 研究への参加をご希望の方には、最後に応募フォームへのリンクをご案内いたします

〈追加質問について〉
このアンケートには「男性の生殖機能に関する認知度」に関する追加質問があります。
追加質問は任意でさらに1〜2分程度お時間がかかります。
お時間の許す方は、ぜひ追加質問にもご協力をお願いいたします。

ご自身の率直なお考えをぜひお聞かせください。

* 必須の質問です

1。 2024年11月〜2025年1月に実施された「男性の健康（アンケート・精液検査・尿検査）に関する研究」が行われていたことをご存じでしたか *

*1 つだけマークしてください。*

- 研究が行われていること自体知らなかった　*質問 2 にスキップします*
- 研究が行われていたことは知っていたが内容はあまり理解していなかった　*質問 2 にスキップします*
- 研究内容をある程度知っていたが参加しなかった　*質問 2 にスキップします*
- 実際に研究に参加した　*質問 6 にスキップします*
- 参加しようと考えたが、最終的に参加しなかった　*質問 2 にスキップします*

続いて以下の質問にお答えください

2。 精液検査に参加しなかった理由を教えてください（複数選択可） *

*当てはまるものをすべて選択してください。*

- [ ] 研究の実施自体知らなかった
- [ ] 研究の手続きを見逃した・タイミングを逃した
- [ ] 内容は知っていたが、恥ずかしい・抵抗があった
- [ ] 精液を採取すること自体に抵抗があった
- [ ] 検査結果を知るのが怖かった
- [ ] 家族やパートナーに知られたくなかった
- [ ] 職場に知られるのが心配だった
- [ ] 参加方法や研究内容がよく分からなかった
- [ ] 精子を採取する場所や方法に不安があった
- [ ] 忙しくて時間がとれなかった
- [ ] 時間帯・場所が合わなかった
- [ ] 面倒だと感じた
- [ ] 自分には関係ないと思った
- [ ] 将来的に子どもを望んでいないから必要を感じなかった
- [ ] 周囲が参加していなかったので気が進まなかった
- [ ] その他: ____________________

3。 生殖能力について、もっと情報があれば知りたいと思いますか？（単一選択） *

*1 つだけマークしてください。*

- ◯ はい
- ◯ どちらともいえない
- ◯ いいえ

4。 今後、精液検査や同様の研究に協力してもよいと思いますか？（単一選択） *

*1 つだけマークしてください。*

- 協力したい　*質問 5 にスキップします*
- 条件が合えば協力してもよい　*質問 5 にスキップします*
- わからない　*質問 5 にスキップします*
- 協力するつもりはない　*質問 5 にスキップします*

*質問 5 にスキップします*

ここから先は男性の健康（生殖機能を含む）に関する詳しいご意見を伺う追加質問です（所要時間1～2分）

5。 ご協力いただけますか？ *

*1 つだけマークしてください。*

- はい　*質問 6 にスキップします*
- いいえ　*セクション 5（）にスキップ*

6。 年齢を教えてください *

*1 つだけマークしてください。*

- 29歳以下
- 30－39歳
- 40－49歳
- 50歳以上

7。 精子の状態が、男性の全般的な健康に影響を与える可能性があると聞いたことがありますか *

*1 つだけマークしてください。*

- よく知っている
- なんとなく聞いたことがある
- ほとんど知らなかった

8。 将来子供を望むうえで「男性の健康」が重要だと思いますか *

*1 つだけマークしてください。*

- 非常に重要だと思う
- ある程度重要だと思う
- どちらともいえない
- あまり重要だと思わない
- 全く重要だと思わない

9。 精液検査を受けることにどの程度心理的抵抗を感じますか *

*1 つだけマークしてください。*

- 非常に強く感じる
- ある程度感じる
- どちらともいえない
- あまり感じない
- 全く感じない

10。 精液検査の結果が「良くない」と判明することにどの程度不安がありますか *

*1 つだけマークしてください。*

- 非常に不安を感じる
- ある程度不安を感じる
- どちらともいえない
- あまり不安を感じない
- 全く不安を感じない

11。 採取場所やプライバシー保護についてどの程度不安がありますか *

*1 つだけマークしてください。*

- 非常に不安に感じる
- ある程度不安に感じる
- どちらともいえない
- あまり不安を感じない
- 全く不安を感じない

12。 男性が精液検査を受けることに対して周囲（家族、パートナー、友人）はどのように反応すると思いますか *

*1 つだけマークしてください。*

- 大きな抵抗感があると思う
- 少し抵抗感があると思う
- 何も感じないと思う
- 支持してくれると思う
- 分からない

13。 もし費用や場所、採取方法がもっと簡単になったら精液検査を受けたいと思いますか *

*1 つだけマークしてください。*

- はい
- いいえ
- わからない

14。 最後にご意見・ご感想・その他、男性の健康や生殖機能に関して感じていることがあれば自由にご記入ください

アンケートは以上で終了になります。

（送信ボタンを押してください）

このコンテンツは Google が作成または承認したものではありません。

Google フォーム
