# Supplementary material for: Very Low Uptake in Workplace Semen Analysis Research: Formative Web-Based Cross-Sectional Follow-Up Survey Distinguishing Employees With Self-Reported Unawareness From Aware Nonparticipants
Source: JMIR Form Res. 2026 Jul 13;10:e90788. doi: 10.2196/90788 (PMC13361622; doi:10.2196/90788)
Supplement: Multimedia Appendix 3 [file formative-v10-e90788-s003.docx]

**Multimedia Appendix 3. Questionnaire (English translation)**

**Health Questionnaire for Men Working in Manufacturing**

Note: The questionnaire was administered in Japanese. The following is an English translation provided for transparency and reproducibility.

This questionnaire is for men working in manufacturing. It asks about your participation in the study on men’s health (questionnaire, semen analysis, and urinalysis) conducted between November 2024 and January 2025 and about your views regarding your own health.

• All responses are anonymous and voluntary (it takes about 2–3 minutes to complete).

• Your responses will be used to analyze research data and to improve future survey methods.

• Completion of this questionnaire will be regarded as providing consent to participate in this survey.

• If you wish to participate in the research, a link to the application form will be provided at the end.

[About additional questions]

This questionnaire includes optional additional questions about “awareness of male reproductive function.”

These additional questions are optional and will take an additional 1–2 minutes.

If you have time, we would appreciate your cooperation with the additional questions as well.

Please share your honest thoughts.

* Questions marked with an asterisk (*) are required.

**Q1. (*)** Did you know that the “study on men’s health (questionnaire, semen analysis, and urinalysis)” conducted between November 2024 and January 2025 was being carried out? (Single choice)

Please select one.

- I was not aware that the study was being conducted. [Skip to Question 2.]
- I knew the study was being conducted, but I did not really understand the details. [Skip to Question 2.]
- I knew the study details to some extent, but I did not participate. [Skip to Question 2.]
- I actually participated in the study. [Skip to Question 6.]
- I considered participating but ultimately did not participate. [Skip to Question 2.]

**Q2. (*)** Please tell us the reasons you did not participate in the semen analysis. (Multiple choice (select all that apply))

Please select all that apply.

- I was not aware that the study was being conducted.
- I missed the study procedures / I missed the timing.
- I knew about the study, but I felt embarrassed or reluctant to participate.
- I felt reluctant to collect a semen sample.
- I was afraid to know the test results.
- I did not want my family or partner to know.
- I was worried that people at my workplace would find out.
- I did not understand the participation method or the study content well.
- I was concerned about the location or method of providing the semen sample.
- I was busy and could not make time.
- The time and/or location did not work for me.
- It felt like a hassle.
- I thought it was not relevant to me.
- I do not want children in the future, so I did not feel it was necessary.
- I was not motivated because people around me were not participating.
- Other: ________

**Q3. (*)** If more information were available about reproductive ability, would you like to know more? (Single choice)

Please select one.

- Yes
- Neither / Unsure
- No

**Q4. (*)** In the future, would you be willing to cooperate with semen analysis or similar research studies? (Single choice)

Please select one.

- I would like to cooperate. [Skip to Question 5.]
- I would cooperate if conditions are met. [Skip to Question 5.]
- I do not know. [Skip to Question 5.]
- I do not intend to cooperate. [Skip to Question 5.]

*From here onward are additional questions asking for your detailed opinions about men’s health (including reproductive function). (Estimated time: 1–2 minutes)*

**Q5. (*)** Would you be willing to answer the additional questions? (Single choice)

Please select one.

- Yes [Skip to Question 6.]
- No [Skip to Section 5 (final comments section).]

**Q6. (*)** Please tell us your age. (Single choice)

Please select one.

- 29 years or younger
- 30–39 years
- 40–49 years
- 50 years or older

**Q7. (*)** Have you ever heard that sperm condition may affect men’s overall health? (Single choice)

Please select one.

- I know this well.
- I have heard something about it.
- I did not know (almost at all).

**Q8. (*)** When hoping to have children in the future, do you think “men’s health” is important? (Single choice)

Please select one.

- I think it is very important.
- I think it is important to some extent.
- Neither / Unsure
- I do not think it is very important.
- I do not think it is important at all.

**Q9. (*)** How much psychological resistance do you feel toward undergoing semen analysis? (Single choice (5-point scale))

Please select one.

- I feel very strongly.
- I feel to some extent.
- Neither / Unsure
- I do not feel much.
- I do not feel at all.

**Q10. (*)** How anxious are you about finding out that your semen analysis results are “not good”? (Single choice (5-point scale))

Please select one.

- I feel very anxious.
- I feel anxious to some extent.
- Neither / Unsure
- I do not feel much anxiety.
- I do not feel anxious at all.

**Q11. (*)** How anxious are you about the collection location and/or privacy protection? (Single choice (5-point scale))

Please select one.

- I feel very anxious.
- I feel anxious to some extent.
- Neither / Unsure
- I do not feel much anxiety.
- I do not feel anxious at all.

**Q12. (*)** How do you think people around you (family, partner, friends) would react to a man undergoing semen analysis? (Single choice)

Please select one.

- I think they would feel strong resistance.
- I think they would feel slight resistance.
- I think they would feel nothing in particular.
- I think they would be supportive.
- I do not know.

**Q13. (*)** If the cost, location, and collection method became easier, would you want to undergo semen analysis? (Single choice)

Please select one.

- Yes
- No
- I do not know.

**Q14.** Finally, if you have any opinions, impressions, or other comments regarding men’s health or reproductive function, please write them freely below. (Free text)

*End of questionnaire. (Please press the Submit button.)*
