# Supplementary material for: Very Low Uptake in Workplace Semen Analysis Research: Formative Web-Based Cross-Sectional Follow-Up Survey Distinguishing Employees With Self-Reported Unawareness From Aware Nonparticipants
Source: JMIR Form Res. 2026 Jul 13;10:e90788. doi: 10.2196/90788 (PMC13361622; doi:10.2196/90788)
Supplement: Checklist 1 [file formative-v10-e90788-s004.docx]

**Checklist 1. CHERRIES Checklist (Completed)**

**Associated manuscript:** Very Low Uptake in Workplace Semen-Analysis Research: Distinguishing Employees With Self-Reported Unawareness From Aware Nonparticipants in a Formative Web-Based Follow-up Survey

Note: Items that cannot be applied or were not performed are marked as “Not applicable” or “Not performed”.

| **Section / Item** | **CHERRIES Checklist Item** | **Manuscript Location** | **Description as Reported in Manuscript** |
| --- | --- | --- | --- |
| Design | Describe survey design | Methods > Study Design and Reporting Standards; Methods > Setting and Recruitment (Parent Study Context) | Formative, anonymous, web-based cross-sectional survey of male employees who had been eligible for, but had not completed, a workplace study involving semen analysis. The survey targeted approximately 900 male employees from 1 cooperating company. Because landing-page visitors and questionnaire starts were unavailable, formal view and participation rates were not reported; the number of analyzable submitted questionnaires and the optional Part 2 completion proportion were reported instead. |
| IRB approval and informed consent process | IRB approval | Methods > Ethical Considerations | Protocol approved by the Institutional Ethics Committee as an amendment to the original project (Approval No. 24-TA-055); conducted in accordance with the Declaration of Helsinki. |
| IRB approval and informed consent process | Informed consent | Ethical Considerations; Methods > Survey Development, Structure, and Measures | Participation was strictly voluntary. After viewing the brief explanation on the landing page, respondents who proceeded to and submitted the questionnaire were considered to have provided electronic informed consent. |
| IRB approval and informed consent process | Data protection | Methods > Survey Development, Structure, and Measures | No personally identifiable information collected; research team did not collect/access/analyze IP addresses or server logs; data stored in Google Forms (HTTPS); access to raw dataset restricted to investigators. |
| Development and pre-testing | Development and testing | Survey Development, Structure, and Measures | The questionnaire was developed by the research team based on experiences from the parent study and previous literature on barriers to male participation in fertility research and research participation more broadly. Draft items underwent multidisciplinary expert review for face validity and clarity, but formal pilot testing and cognitive interviewing were not performed. The questionnaire was therefore considered a study-specific preliminary instrument for formative description rather than a validated measure of underlying constructs. |
| Recruitment process and sample access | Open survey versus closed survey | Setting and Recruitment (Parent Study Context) | Closed target population in the sense that the survey targeted approximately 900 male employees from 1 cooperating company. Access was initiated offline through business card–sized flyers distributed during routine health checkups, with a QR code linking directly to a Google Form. |
| Recruitment process and sample access | Contact mode | Methods > Setting and Recruitment (Parent Study Context) | Initial contact was offline (business card-sized flyers distributed during routine health checkups); participation via QR code to Google Forms. |
| Recruitment process and sample access | Advertising the survey | Setting and Recruitment (Parent Study Context) | Survey invitations were distributed as business card–sized flyers during routine health checkups and included a QR code linking directly to a Google Form. The exact flyer wording is not reported in the current main text. |
| Survey administration | Web/E-mail | Methods > Setting and Recruitment (Parent Study Context); Methods > Survey Development, Structure, and Measures | The survey was administered as a web-based Japanese questionnaire using Google Forms; respondents accessed it directly via a QR code on the offline invitation flyer. |
| Survey administration | Context | Methods > Setting and Recruitment (Parent Study Context) | Not applicable: the survey was not posted on a public website or mailing list/newsgroup; it was accessed via QR code on an invitation flyer. |
| Survey administration | Mandatory/voluntary | Methods > Ethical Considerations; Methods > Survey Development, Structure, and Measures | Participation in the survey was strictly voluntary. Within the questionnaire, Part 1 was mandatory, whereas Part 2 was optional and shown only to respondents who chose to continue to the optional section. |
| Survey administration | Incentives | Methods > Ethical Considerations | No financial or other incentives were provided. |
| Survey administration | Time/Date | Methods > Setting and Recruitment (Parent Study Context) | Invitations distributed April–May 2025; parent study recruitment November 2024–January 2025. |
| Survey administration | Randomization of items or questionnaires | Methods > Survey Development, Structure, and Measures | No item randomization was used. |
| Survey administration | Adaptive questioning | Methods > Survey Development, Structure, and Measures | The survey used a 2-part branching structure. Part 1 was mandatory, and Part 2 was optional; respondents who chose to continue to Part 2 saw a second brief explanation before the detailed items. |
| Survey administration | Number of items | Methods > Survey Development, Structure, and Measures; Methods > Statistical Analysis | The questionnaire consisted of 14 items in total. Part 1 (4 structured core items) assessed awareness of the parent study, reasons for nonparticipation, interest in information on male reproductive ability, and general openness toward future semen-analysis or similar research. Following a single gate item, Part 2 (8 optional structured items) assessed age, male reproductive health knowledge, perceived importance of male health for future conception, study-specific self-reported resistance item, anxiety about poor results, concerns about collection location and/or privacy protection, expected reactions of others, and willingness to undergo semen analysis under simplified conditions. An optional free-text field (1 item) was reviewed for contextual understanding but was not analyzed qualitatively. |
| Survey administration | Number of screens (pages) | Methods > Survey Development, Structure, and Measures | The exact number of screens/pages was not reported. The manuscript describes a landing page, a 2-part branching structure, and a second brief explanation before the optional Part 2 items. |
| Survey administration | Completeness check | Methods > Survey Development, Structure, and Measures; Results > Survey Participation, Completion, and Data Quality | Google Forms required-response settings were enabled for all questions, and data screening identified no missing values. |
| Survey administration | Review step | Methods > Survey Development, Structure, and Measures | Respondents could review and change their answers before submission. |
| Response rates | Unique site visitor | Methods > Survey Development, Structure, and Measures | Not applicable/not available: invitations were distributed offline, and the questionnaire was accessed directly via QR code without prospectively configured web analytics or server-log tracking. IP addresses and platform server logs were not accessed; therefore, unique visitors could not be determined. |
| Response rates | View rate (unique survey visitors / unique site visitors) | Methods > Setting and Recruitment (Parent Study Context) | Not reported: no formal view rate was reported because the number of landing-page visitors was unavailable. |
| Response rates | Participation rate (agreed to participate / first survey page visitors) | Setting and Recruitment (Parent Study Context); Results > Survey Participation, Completion, and Data Quality | Not reported: no formal participation rate was reported because the numbers of landing-page visitors and questionnaire starts were unavailable. Instead, the manuscript reports the number of analyzable submitted questionnaires and the completion proportion for optional Part 2. |
| Response rates | Completion rate (finished / agreed to participate) | Results > Survey Participation, Completion, and Data Quality | The formal CHERRIES completion rate from agreed participants/questionnaire starters could not be determined because the number of questionnaire starts was unavailable. Among 108 valid respondents who completed Part 1, 83 completed the optional detailed section, yielding an optional Part 2 completion proportion of 76.9%. |
| Preventing multiple entries | Cookies used | Methods > Survey Development, Structure, and Measures | Not used/not analyzed: no cookie-based or other technical duplicate-prevention mechanism was feasible; no unique technical identifiers were collected or analyzed. |
| Preventing multiple entries | IP check | Methods > Survey Development, Structure, and Measures | Not used: IP addresses were not collected, accessed, or analyzed. |
| Preventing multiple entries | Log file analysis | Methods > Setting and Recruitment (Parent Study Context); Methods > Survey Development, Structure, and Measures | Not used: platform server logs were not accessed, and the survey was conducted without prospectively configured server-log tracking. |
| Preventing multiple entries | Registration | Methods > Survey Development, Structure, and Measures | Not applicable: the survey was accessed directly via a QR code linking to a Google Form and did not use a registration-based sampling frame. |
| Analysis | Handling of incomplete questionnaires | Methods > Survey Development, Structure, and Measures; Methods > Statistical Analysis; Results > Survey Participation, Completion, and Data Quality | Required-response settings minimized item nonresponse, and data screening identified no missing values. Analyses of Part 1 used all 108 valid respondents, whereas analyses of Part 2 variables were restricted to the 83 respondents who completed the optional section. |
| Analysis | Questionnaires submitted with an atypical timestamp | Methods > Survey Development, Structure, and Measures; Results > Survey Participation, Completion, and Data Quality | Not performed as a formal exclusion rule: no completion-time cut-off was specified. The dataset was screened manually for obvious duplicates, such as identical response patterns with the same timestamp, and no obvious duplicate entries were identified. |
| Analysis | Statistical correction | Methods > Statistical Analysis | Not performed/not reported: analyses were descriptive, 95% CIs for proportions were calculated using the Wilson score method, and no formal hypothesis testing, weighting, propensity-score adjustment, or other correction for non-representativeness was reported. |
