# Supplementary material for: Very Low Uptake in Workplace Semen Analysis Research: Formative Web-Based Cross-Sectional Follow-Up Survey Distinguishing Employees With Self-Reported Unawareness From Aware Nonparticipants
Source: JMIR Form Res. 2026 Jul 13;10:e90788. doi: 10.2196/90788 (PMC13361622; doi:10.2196/90788)
Supplement: Checklist 2 [file formative-v10-e90788-s005.docx]

**Checklist 2. CROSS Checklist (Completed)**

**Associated manuscript:** Very Low Uptake in Workplace Semen-Analysis Research: Distinguishing Employees With Self-Reported Unawareness From Aware Nonparticipants in a Formative Web-Based Follow-up Survey

Note: Items that cannot be applied or were not performed are marked as “Not applicable” or “Not performed”.

| **Section / Item** | **CROSS Checklist Item** | **Manuscript Location** | **Description as Reported in Manuscript** |
| --- | --- | --- | --- |
| Title and abstract (1a) | State the word “survey” along with a commonly used term in title or abstract to introduce the study’s design. | Title; Abstract | The Title includes the word “Survey” (specifically “Web-Based Follow-up Survey”). The Abstract explicitly specifies the study design as an “anonymous web-based cross-sectional follow-up survey.” |
| Title and abstract (1b) | Provide an informative summary in the abstract, covering background, objectives, methods, findings/results, interpretation/discussion, and conclusions. | Abstract | Abstract includes Background, Objectives, Methods, Results with key counts and proportions, and Conclusions. The Methods state that Wilson 95% CIs were calculated for proportions; detailed 95% CIs are provided in the Results tables. |
| Introduction – Background (2) | Provide background about the rationale of study, what has been previously done, and why this survey is needed. | Introduction | Introduces recruitment challenges in semen-analysis research, the occupational and ethical rationale for workplace-based reproductive health research, and the need to distinguish limited recruitment reach from limited acceptability among previously invited nonparticipants. |
| Introduction – Purpose/aim (3) | Identify specific purposes, aims, goals, or objectives of the study. | Abstract > Objectives; Introduction (last paragraph) | States the aim to describe reasons for nonparticipation in a workplace semen-analysis study and to explore whether these reasons differed between previously unreached workers and aware nonparticipants. |
| Methods – Study design (4) | Specify the study design in the “Methods” section with a commonly used term (e.g., cross-sectional or longitudinal). | Methods > Study Design and Reporting Standards | Describes a formative, anonymous, web-based cross-sectional survey. |
| Methods – Data collection methods (5a) | Describe the questionnaire (e.g., number of sections, number of questions, number and names of instruments used). | Methods > Survey Development, Structure, and Measures | Questionnaire used a two-part branching structure: Part 1 was mandatory and assessed awareness of the parent study, reasons for nonparticipation, interest in male reproductive health information, and general openness to future semen-analysis or similar research; Part 2 was optional and assessed age, reproductive health knowledge, study-specific self-reported resistance item, anxiety about unfavorable results, concerns about collection location and/or privacy protection, expected reactions from others, and willingness under simplified conditions. |
| Methods – Data collection methods (5b) | Describe all questionnaire instruments used to measure concepts; report target population, validity/reliability information, scoring/classification procedure, and references (if any). | Methods > Survey Development, Structure, and Measures; Methods > Statistical Analysis | Custom (non-validated) items developed by study team; face validity reviewed by multidisciplinary experts; Likert responses aggregated for interpretation (e.g., top two categories combined) and other categories grouped conceptually; study-specific preliminary instrument rather than a validated measure of underlying constructs. |
| Methods – Data collection methods (5c) | Provide information on pretesting of the questionnaire, if performed (method, frequency, participant demographics, similarity to sample). | Methods > Survey Development, Structure, and Measures | Pretesting not performed: draft items underwent multidisciplinary expert review for face validity and clarity, but formal pilot testing and cognitive interviewing were not performed. |
| Methods – Data collection methods (5d) | Questionnaire, if possible, should be fully provided (in the article, appendices, or online supplement). | Methods > Survey Development, Structure, and Measures | The complete original Japanese questionnaire, provided as a PDF export of the Google Form used for data collection, is included as Multimedia Appendix 4. An English translation of all items and response options is provided as Multimedia Appendix 5. |
| Methods – Sample characteristics (6a) | Describe study population (background, locations, inclusion/exclusion criteria). | Methods > Study Design and Reporting Standards; Methods > Setting and Recruitment (Parent Study Context); Results > Survey Participation, Completion, and Data Quality | Targeted male employees in Japan who had been eligible for, but had not completed, the parent workplace semen-analysis study. The present survey was conducted among approximately 900 male employees from one cooperating company, and no respondent selected the option indicating actual participation in the parent study. |
| Methods – Sample characteristics (6b) | Describe sampling techniques used (e.g., convenience sampling); specify clustered locations when applicable. | Methods > Setting and Recruitment (Parent Study Context) | Convenience sample of employees invited via flyers distributed during routine health checkups; closed corporate setting (one company for this additional survey). |
| Methods – Sample characteristics (6c) | Provide information on sample size and sample size calculation. | Methods > Setting and Recruitment (Parent Study Context); Results > Survey Participation, Completion, and Data Quality | Invited approximately 900 employees; obtained 108 valid responses; no a priori sample size calculation reported (formative/descriptive survey). The parent study minimum target was a pragmatic feasibility threshold, not a formal power calculation; the present survey was formative/descriptive. |
| Methods – Sample characteristics (6d) | Describe how representative the sample is of the study population/target population (if possible). | Methods > Setting and Recruitment (Parent Study Context); Results > Survey Participation, Completion, and Data Quality; Limitations | Representativeness is limited because the study used a single-company convenience sample of nonparticipants, the exact denominator/view/start rates were unavailable, and Part 2 was completed by a self-selected subgroup. Findings are interpreted as formative and preliminary rather than as population estimates. |
| Methods – Survey administration (7a) | Provide modes of questionnaire administration, type/number of contacts, and location where survey was conducted. | Methods > Setting and Recruitment (Parent Study Context); Methods > Survey Development, Structure, and Measures | Offline invitations were distributed as business card–sized flyers during routine health checkups; the survey was completed online via Google Forms accessed directly through a QR code. |
| Methods – Survey administration (7b) | Provide survey time frame (recruitment/exposure/follow-up days). | Methods > Setting and Recruitment (Parent Study Context) | Survey recruitment and data collection occurred April–May 2025; parent study recruitment November 2024–January 2025. |
| Methods – Survey administration (7c) | Provide entry process; for web-based surveys, approaches to prevent multiple participation. | Methods > Setting and Recruitment (Parent Study Context); Methods > Survey Development, Structure, and Measures | Access was via QR code directly to a Google Form. No prospective web analytics or server-log tracking was configured, IP addresses and platform server logs were not accessed, and technical prevention of duplicate entries was not feasible. The dataset was screened manually for obvious duplicates, such as identical response patterns with the same timestamp. |
| Methods – Study preparation (8) | Describe preparation process before conducting survey (e.g., advertising, training). | Methods > Survey Development, Structure, and Measures; Methods > Setting and Recruitment (Parent Study Context) | The questionnaire was developed based on the parent study experience and previous literature, followed by multidisciplinary expert review for face validity and clarity. Invitation flyers with QR codes were prepared for offline distribution during routine health checkups. Formal pilot testing and cognitive interviewing were not performed. |
| Methods – Ethical considerations (9a) | Provide information on ethical approval, informed consent, IRB approval, Helsinki/GCP as appropriate. | Methods > Ethical Considerations | Ethics approval was obtained as an amendment to the original project (Approval No. 24-TA-055), the study was conducted in accordance with the Declaration of Helsinki, participation was strictly voluntary, and electronic informed consent was obtained before access to the questionnaire was granted. |
| Methods – Ethical considerations (9c) | Provide information about survey anonymity/confidentiality and mechanisms to protect unauthorized access. | Methods > Ethical Considerations; Methods > Setting and Recruitment (Parent Study Context); Methods > Survey Development, Structure, and Measures | The survey was anonymous. No personal identifiers were collected, IP addresses and platform server logs were not accessed, invitations were distributed offline to reduce workplace surveillance concerns, no identifiable data were shared with companies, and the cooperating companies had no role in data analysis or interpretation. |
| Methods – Statistical analysis (10a) | Describe statistical methods/analytical approach and software. | Methods > Statistical Analysis | Descriptive statistics; Wilson score 95% CIs; aggregation of response categories for interpretability; analyses performed in R (version 4.3.1). |
| Methods – Statistical analysis (10b) | Report any modification of variables used in analysis, with references if available. | Methods > Statistical Analysis | Likert responses collapsed (top two categories combined) and categories aggregated conceptually for some items; rationale cited. |
| Methods – Statistical analysis (10c) | Report how missing data was handled (rate, mechanism, methods). | Methods > Survey Development, Structure, and Measures; Results > Survey Participation, Completion, and Data Quality; Methods > Statistical Analysis | All questions used Google Forms required-response settings, and no missing values were present among submitted responses. Analyses of Part 2 variables were restricted to respondents who completed the optional section; no imputation was performed. |
| Methods – Statistical analysis (10d) | State how non-response error was addressed. | Not applicable | No formal response rate, view rate, or participation rate was reported because the exact numbers of employees who received, viewed, or started the survey were unavailable. Potential non-response and selection bias are discussed, and findings are interpreted as formative rather than population estimates. |
| Methods – Statistical analysis (10e) | For longitudinal surveys, state how loss to follow-up was addressed. | Not applicable | Not applicable: study was cross-sectional with no follow-up. |
| Methods – Statistical analysis (10f) | Indicate whether weighting/propensity scores used to adjust for non-representativeness. | Methods > Statistical Analysis | Not performed: no weighting/propensity score adjustments; findings interpreted descriptively. |
| Methods – Statistical analysis (10g) | Describe any sensitivity analysis conducted. | Methods > Statistical Analysis; Results > Table 4; Results > Table 5 | Post-hoc awareness-stratified analyses were conducted. A secondary descriptive sensitivity analysis divided the aware stratum into “aware but poorly informed” and “aware and declined” groups, and exploratory awareness-stratified analyses of optional Part 2 items were conducted among completers. These analyses were descriptive and interpreted cautiously. |
| Results – Respondent characteristics (11a) | Report numbers of individuals at each stage; consider flow diagram. | Results > Survey Participation, Completion, and Data Quality | Reported approximate invited (≈900), , analyzable submitted questionnaires (n=108), and optional Part 2 completions (n=83). No flow diagram included in main text. |
| Results – Respondent characteristics (11b) | Provide reasons for non-participation at each stage, if possible. | Results > Post-hoc Stratified Analysis of Reasons for Nonparticipation; Results > Table 2; Results > Table 4 | Reasons for nonparticipation in the parent study are reported in the awareness-stratified analysis in Table 2, with a secondary 3-strata descriptive sensitivity analysis in Table 4. Reasons for not proceeding from Part 1 to optional Part 2 were not collected. |
| Results – Respondent characteristics (11c) | Report response rate and define response rate/formula. | Methods > Setting and Recruitment (Parent Study Context); Results > Survey Participation, Completion, and Data Quality | No formal response rate is reported because the exact numbers of employees who received, viewed, or started the survey were unavailable. The manuscript reports approximately 900 employees invited, 108 analyzable submitted questionnaires, and Part 2 completion proportion among respondents. |
| Results – Respondent characteristics (11d) | Define how unique visitors are determined; report unique visitors and proportions (view/participation/completion). | Methods > Setting and Recruitment (Parent Study Context); Methods > Survey Development, Structure, and Measures; Results > Survey Participation, Completion, and Data Quality | Not applicable: invitations were distributed offline, no prospective analytics or server-log tracking was configured, and IP addresses/platform server logs were not accessed. Therefore, unique visitors, view rate, and participation rate could not be calculated. Completion of optional Part 2 among valid respondents was reported as 83/108 (76.9%). |
| Results – Descriptive results (12) | Provide characteristics of participants, potential confounders, and outcomes. | Results > Tables 1–5 | Participant responses are summarized descriptively with counts, percentages, and Wilson 95% CIs: Table 1 summarizes overall Part 1 responses; Table 2 presents awareness-stratified reasons for nonparticipation; Table 3 summarizes Part 2 characteristics, knowledge, psychological barriers, and willingness; Table 4 provides a secondary 3-strata sensitivity analysis; and Table 5 provides exploratory awareness-stratified Part 2 analyses. |
| Results – Main findings (13a) | Give unadjusted (and if applicable adjusted) estimates with 95% CIs and p values. | Abstract; Results > Tables 1–5 | The abstract reports key counts and proportions. The Results tables provide unadjusted descriptive proportions with Wilson 95% CIs. No hypothesis testing, adjusted analyses, or p values were reported because analyses were descriptive. |
| Results – Main findings (13b) | For multivariable analysis, report model-building, fit, assumptions. | Not applicable | Not applicable: no multivariable modeling was performed. |
| Results – Main findings (13c) | Provide details about sensitivity analysis performed (and missing-data sensitivity if relevant). | Results > Table 4; Results > Table 5 | A secondary descriptive sensitivity analysis using 3 awareness strata is reported in Table 4, and exploratory awareness-stratified analyses of optional Part 2 items are reported in Table 5. These analyses were post-hoc/descriptive and should not be interpreted as confirmatory subgroup evidence. Missing-data sensitivity analysis was not required because no missing values were present. |
| Discussion – Limitations (14) | Discuss limitations considering biases and imprecisions. | Limitations | Discusses the single-company convenience sample of nonparticipants, likely selection bias, unavailable exact denominator/view/start rates, optional Part 2 self-selection, use of study-specific items without formal pilot testing or cognitive interviewing, wording/framing limitations, lack of assessment of occupational reproductive hazard awareness or education, and the very small number of parent-study participants preventing direct comparison between participants and nonparticipants. |
| Discussion – Interpretations (15) | Give cautious overall interpretation considering biases and suggest future research. | Discussion > Principal Findings; Discussion > Comparison With Prior Work and Interpretation; Conclusions | Interprets low uptake as reflecting two distinct problems: limited recruitment reach and limited acceptability of semen analysis as a voluntary workplace research procedure. The Discussion emphasizes the formative/descriptive nature of the findings, the denominator problem clarified by awareness stratification, the legitimacy of refusal, and the need for clearer communication, strong privacy protection, exposure-defined research questions, and lower-burden/private participation pathways. |
| Discussion – Generalizability (16) | Discuss external validity. | Limitations; Discussion | Notes limited external validity due to the single-company convenience sample, likely selection bias, unavailable reach metrics, and optional Part 2 self-selection. Findings are intended as formative evidence for similar workplace-based semen-analysis research contexts rather than as population estimates for all invited workers, all nonparticipants, or Japanese men generally. |
| Other sections – Role of the funding source (17) | State whether any funding organization had roles in survey design, implementation, and analysis. | Funding | Funding source is stated as the Japan Science and Technology Agency, Grant Number JPMJPF2017. The manuscript explicitly states that the funder had no role in study design; data collection, analysis, or interpretation; manuscript preparation; or the decision to submit the manuscript for publication. |
| Other sections – Conflict of interest (18) | Declare potential conflicts of interest. | Conflicts of Interest | Authors declare no conflict of interest. |
| Other sections – Acknowledgements (19) | Provide names of organizations/persons acknowledged and their contribution. | Acknowledgments | Acknowledges specific individuals for professional advice and laboratory operations; also discloses use of ChatGPT/Gemini/Perplexity for proofreading and literature assistance. |
